# Effects of glibenclamide and troxerutin on the sperm parameters and histopathological changes of testis in streptozotocin-induced diabetic male rats: An experimental study

**DOI:** 10.18502/ijrm.v21i2.12803

**Published:** 2023-03-08

**Authors:** Aynaz Shokri, Bagher Pourheydar, Gholam Hossein Farjah, Mojtaba Krimipour, Maryam Pourheydar

**Affiliations:** ^1^Faculty of Medicine, Urmia University of Medical Sciences, Urmia, Iran.; ^2^Neurophysiology Research Center, Department of Anatomical Sciences, Faculty of Medicine, Urmia University of Medical Sciences, Urmia, Iran.; ^3^Department of Anatomical Sciences, Faculty of Medicine, Urmia University of Medical Sciences, Urmia, Iran.

**Keywords:** Diabetes, Glibenclamide, Sperm, Testis, Troxerutin.

## Abstract

**Background:**

Oxidative stress is a major contributor to diabetes mellitus (DM), which leads to testicular damage and infertility.

**Objective:**

The aim of this study was to investigate the effects of glibenclamide (GL) as a chemical medicine and troxerutin (TR) as an herbal agent on sperm parameters and histopathological changes of testis in diabetic male rats.

**Materials and Methods:**

Forty male Wistar rats (230-260 gr) were randomly divided into 5 groups (n = 8/each), including control, diabetic (D), GL, TR, and GL+TR. DM was induced by the administration of 60 mg/kg streptozotocin intraperitoneally. The groups were treated with 5 mg/kg/day of GL or 150 mg/kg/day of TR via oral gavage for 4 wk. In the final stage of the treatment, blood sampling was done for biochemical analysis. The rats were then sacrificed and their left testis and epididymis were dissected for sperm analysis, histopathology, and morphometric assessment.

**Results:**

A significant decrease in the number, motility, viability, maturity, and chromatin quality of sperm was found in diabetic rats compared to control group. (p < 0.001). DM also increased the malondialdehyde level and decreased the level of the serum's total antioxidant capacity compared to the control group (p < 0.001). Furthermore, we observed a significant difference in seminiferous tubule diameter, germinal epithelium height, and testicular histological abnormalities in diabetic rats compared to control group (p < 0.001). Administration of GL, TR, and their combination improved the above-mentioned parameters, and treatment with TR provided a higher improvement (p < 0.001).

**Conclusion:**

According to these findings, it can be concluded that TR plays a more influential role than GL to treat diabetic-induced infertility.

## 1. Introduction

Diabetes mellitus (DM) is the most common metabolic and endocrine disorder, and its complications have made it one of the major health problems (1). During DM, hyperglycemia negatively affects various organs, especially the testis (2). Previous research showed that DM causes reproductive dysfunction, including sex organ atrophy, testosterone deficiency, and sperm count and motility loss (3). Oxidative stress (OS) plays an important role in the pathogenesis of DM. It is well known that hyperglycemia increases reactive oxygen species (ROS). Excessive production of ROS leads to alterations in protein, nucleic acids, and phospholipid structures and results in DNA and RNA damages (4). It induces testicular cell apoptosis and decreases the levels of antioxidant enzymes (5).

One of the chemical agents that are widely used for treating DM is glibenclamide (GL). GL is one of the drugs from the sulphonylureas category. It promotes insulin release in pancreatic beta cells by blocking ATP-sensitive potassium channels. GL contains antidiabetic, antinociceptive, antitumor, anti-oxidative, and platelet aggregation inhibitor characteristics, among others. Despite having beneficial properties, GL has several side effects, such as hypoglycemia and gastrointestinal complications. One of the most common side effects of GL is hypoglycemia, which, if left untreated, can lead to seizures, coma, or death. The most common gastrointestinal symptoms are constipation, diarrhea, nausea, vomiting, abdominal pain, and loss of appetite (6). Since chemical drugs have undesirable side effects, herbal plants are considered alternative therapies because of their antioxidant properties and fewer side effects.

Troxerutin (TR), often known as vitamin P4, is a bioflavonoid derivative that has been tri-hydroxymethylated. Coffee, tea, fruits, vegetables, and cereal grains all contain it. TR has antioxidant (7), antihyperglycemic (8), anti-inflammatory (9), anti-thrombolytic, and anti-fatigue (10) activities. TR antioxidant properties may be related to its ability to reduce ROS generation while also activating antioxidant enzymes (11). Furthermore, TR improves insulin sensitivity in cells (12). Some studies have been confirmed on GL's and TR's anti-hyperglycemic and antioxidative effects (6, 7). Another study has indicated that although GL is an effective antihyperglycemic agent used for the treatment of DM; however, it is a chemical drug and possesses some side effects that negatively affect the patient's quality of life. It is believed that chemical medications have more side effects than herbal agents (6).

Therefore, this study aimed to compare the effects of TR (an herbal material) with those of GL (a chemical drug) on the testicular tissue and sperm parameters in streptozotocin-induced diabetic rats. To the best of the authors' knowledge, there is no study comparing the effects of GL and TR on sperm parameters and testicular tissue in diabetic rats. Given the beneficial effects of TR on the male reproductive system, it is believed that the findings of this research will serve as a guideline for reducing diabetic-induced deleterious effects on sperm parameters and testicular structure, as well as be useful in the treatment of infertility.

## 2. Materials and Methods

### Animals

In this experimental study, 40 male Wistar rats weighing 230-260 gr (10-14 wk old) were obtained from the animal house of the Medicine at Urmia University of Medical Sciences, Urmia, Iran. Animals were maintained under a controlled room temperature of 21-23C with a 12-hr light/dark cycle and a humidity level of 50-60%. All animals had access to water and food ad libitum. This study was performed in 2020 at Urmia University of Medical Sciences, Urmia, Iran.

### Experimental design

The animals were randomly divided into 5 groups (n = 8/each), and the drugs were administered daily through oral gavage for 4 wk: 1) Control (C) group received an equal volume of saline via oral gavage, 2) Diabetic (D) group received streptozotocin (STZ, Sigma, St. Louis, Mo, USA) (60 mg/kg/IP) (13), 3) TR group received STZ+TR (Merck, Germany) (150 mg/kg/po) (14), 4) GL group received STZ+GL (Sigma-Aldrich Chemie, Steinheim, Germany) (5 mg/kg/po) (15), and 5) GL+TR group received STZ, TR, and GL.

### Induction of DM

A single injection of STZ-induced DM (60 mg/kg/IP), was freshly dissolved in normal cold saline. 3 days after STZ injection, blood samples were taken from the tail vein and glucose measured by a digital glucometer (Elegance, Model: no: CT-X10 Germany). Rats with blood glucose levels > 250 mg/dl were acknowledged as diabetic.

In the case of death, the animals were excluded from the study. In this study, 3 diabetic animals died and were replaced by live diabetic animals. Finally, 40 rats were included in the study.

### Epididymal sperm preparation

Sperms were collected from the caudal part of the epididymis (16). All rats were euthanized under anesthesia by an overdose of ketamine (160 mg/kg, Daroopakhsh, Iran) and xylazine (20 mg/kg, Daroopakhsh, Iran). After sterilizing the abdominal skin with 70% ethanol, an incision was made in the abdomen, and the testis with epididymis was removed. Then, the caudal epididymis was separated and placed in 2 ml of warm human tubal fluid (37 C). Then several incisions were made on the epididymis to release more sperm. Finally, the sperm suspension was placed in an incubator at 37 C and 5% CO ${}_{2}$ for 30 min (17).

### Sperm parameters

Counting of the epididymal sperm was performed by a hemocytometer (Neubauer chamber, Deep 1/10 m; LABART, Munich, Germany). After dilution of sperm suspension to (1:20) in human tubal fluid medium, 10 µl of diluted specimen was transferred to the counting chambers of the hemocytometer and counted under a light microscope after 5 min. Sperm count was performed according to the World Health Organization guidelines. The following formula was used to calculate the count of sperm in 1 ml sperm suspension: spermcellnumber=[n. 50,000. d] where n = the counted sperm number, d = reverse of sperm suspension dilution (18).

Eosin-nigrosin staining was used to evaluate sperm viability. A 20 µl of eosin solution was added into the same value of sperm suspension and after 30 sec 20 µl of nigrosin solution was added. After preparing the smears, slides were dried and evaluated under a light microscope (Nikon ECLIPSE E200, Japan) with a magnification of 400X. The percentage of unstained alive sperms and pink stained dead sperms were determined. In this process, at least 200 sperms were counted, and the cell viability was calculated as the percentage of live sperms.

Eosin-nigrosin stained slides were used to evaluate morphology of sperm. One drop of eosin/nigrosine was added to the sperm suspension and mixed mildly. The slides were then investigated under a light microscope at 400X. The existence of one or more abnormal characteristics, such as flaws in the head, tail, or center piece were investigated. A total of 200 sperms were examined for morphological flaws. The results were expressed as a proportion of morphologically normal sperm. For sperm motility assessment, 10 µl of sperm suspension was placed on a microscopic slide. Using a light microscope (Olympus, BH2, Japan) with a magnification of 400X, the number of sperms with progressive forward movement and motionless sperms were counted in several microscopic fields, and the percentage of motile and immobile sperms was calculated.

### Sperm nuclear maturity 

At the spermatogenesis stage in chromatin of sperms, histones are replaced by protamines. This replacement is very important for the sperm's stability and density. Therefore, in order to determine sperm chromatin condensation and assessment of sperm nuclear maturity, aniline blue (AB) staining was used.

A lot of lysine amino acids participate in histone protein structure, which reacts with acidic dyes such as AB. Therefore, in AB staining, dark blue sperms had excessive histone and considered sperms with immature nuclei and light blue sperms had normal chromatin structure and considered healthy sperms. In brief, a drop of sperm suspension was placed on the glass slide and allowed to dry with air, and then the smears were fixed in 3% glutaraldehyde for 30 min.

The smears were then stained with 5% aqueous AB solution for 5 min and incubated in 3% acetic acid for 5 min. Finally, the slides were washed and observed under a light microscope using 100x magnification. In this test, abnormal immature sperms were dark blue in color whereas normal mature sperms were pale. At least 200 sperms were counted in each slide and the data were expressed as a percentage.

### Body weight and testis weight 

For each animal, body weight was measured a day before STZ-injection and at the end of treatment. At the end of the experimental period, the rats were anesthetized with ketamine and xylazine; blood samples were taken from the heart of each animal for malondialdehyde (MDA) and total antioxidant capacity (TAC) levels measurement. Then the rats' testicles were removed, the weight of right testis was recorded and its weight index was calculated. The left testis was used for histological assessment.

### Measurement of serum MDA 

To assess the MDA, blood samples from each rat were obtained from the heart and were collected in sterile tubes. Then the samples centrifuged at (3000 rpm for 15 min) to obtain serum and serum samples were stored at -70 C. To assay the amount of lipid peroxidation, serum levels of MDA were measured. MDA is the production of lipid peroxidation and is used as an index of OS. In brief, the amount of serum level of MDA was determined by placing 0.20 ml of the serum into a test tube containing 3 ml of glacial acid. Then 1% thiobarbituric acid in 2% NaOH was added to the tube. The solution was placed in boiling water for 15 min. After cooling, a spectrophotometer measured the pink-colored product's absorbance at 532 nm. MDA tetrabutylammonium salt, procured from Sigma Company, was used to create the calibration curve.

### Measurement of serum TAC

Measurement of serum TAC was used to determine the protective effects of GL and TR on diabetic-induced OS. Serum levels of TAC was measured according to the guideline and protocol of a commercial kit LDN (Labor Diagnostika Nord GmbH, Germany). This method is based on the reduction ability of Fe ${}^{3+}$ . In this procedure, a large amount of Fe ${}^{3+}$ was converted to Fe ${}^{2+}$ in an acidic pH, resulting in the formation of blue dye. The serum levels of TAC were measured by spectrophotometry and the absorbance of the samples was measured at a wavelength of 593 nm. The amount of TAC was expressed in (nmol/ml) (17, 19).

### Histopathological assay

After excision of the left testis, it was placed in 10% formalin for 48 hr to fix its tissue. Tissue processing was performed and 5-µm thick sections were prepared with a rotary microtome (Leica Model RM 2145, Germany). After staining of slides with hematoxylin and eosin (H&E) (Merk, Germany), they were investigated under a light microscope (Olympus, BH2, Japan) equipped with a SONY on-board camera (Zeiss, Cyber-Shot, Japan) (20). The number of spermatogonia, primary spermatocyte, spermatid, Leydig, and Sertoli cells were counted in different fields, and the mean numbers were calculated.

### Morphometric study

Using the Motic camera and software, the height of the germinal epithelium and the diameter of the seminiferous tubule were measured at random in 50 round or nearly round cross-sections of seminiferous tubules in each group, and the means were determined (21). The diameter of seminiferous tubules was measured in micrometers.

### Apoptosis assay

The percentage of apoptotic cells in testis was identified by TUNEL (terminal deoxynucleotidyl transferase dUTP nick end labeling) staining. This staining was performed using the instructions of the “in situ cell death detection kit” (Roche In situ Death Detection Kit, Germany) (22).

The percentage of TUNEL positive sperms was identified by DNA fragmentation index (DFI). The following formula was used to calculate DFI:

DFI = [TUNEL positive/TUNEL positive + TUNEL negative)] × 100%) (23).

### Ethical considerations

All animal experimentation protocols were carried out under the supervision of the Ethics Committee of Urmia University of Medical Sciences, Urmia, Iran (Code: IR.UMSU.REC.1399.077).

### Statistical analysis

All data were presented as (Means ± SD). Analysis of variance (ANOVA) was applied for multiple group comparison, followed by Tukey's post hoc test. P < 0.05 was considered statistically significant. The statistical analysis was performed using SPSS software version 16.0 for windows (SPSS Inc, Chicago, II).

## 3. Results

### Sperm count 

According to the results, a significant decrease was observed in sperm count of diabetic rats compared to the control group. Based on the results a significant increase was observed in GL, TR, and (GL+TR) groups compared to the diabetic group (p < 0.001). A significant difference in sperm count between the GL group with TR, and GL+TR groups (p < 0.001) was observed (Table I).

### Sperm motility 

Animals in the diabetic group showed a significant decrease in the percentage of motile sperm compared to the control group (p < 0.001). Rats treated with GL and TR showed a remarkable and significant increase in the percentage of motile sperm compared to the diabetic group (p < 0.001). According to the data, a significant difference between GL with TR and (GL+TR) groups (p < 0.001) was observed (Table II).

### Sperm morphology

According to table I, the rats in the diabetic group have a lower rate of sperm with normal morphology compared to the control group. There was a significant decrease in the percentage of sperm with normal morphology in diabetic group compared to the control group (p < 0.001). A significant difference was seen in the percentage of sperm with normal morphology between diabetic rats and those in the GL, TR, and GL+TR groups (p < 0.001). The morphological observations indicated that combination therapy with GL and TR significantly (p < 0.001) improved sperm morphological anomalies and increased the percentage of normal sperms compared to the untreated group so that there was no significant difference between the control and (GL+TR) groups (p = 0.053).

### Sperm viability

According to the results, the percentage of live sperm in the diabetic rats decreased significantly (p < 0.001) compared to those in the control group. When compared to the diabetic group, diabetic rats treated with GL, TR, and their combination showed a considerable increase (p < 0.001) in the percentage of live sperms. Statistical analysis indicated that all treated groups showed a significant increase (p < 0.001) in the percentage of live sperms (Table I).

### Sperm nuclear maturity

The findings of AB staining indicated that the percentage of dark blue stained sperms (sperms with an immature nucleus) was significantly increased in diabetic rats in comparison with control group (p < 0.001) (Table I). Statistical analysis revealed that TR administration improved chromatin abnormalities and significantly decreased the percentage of sperms with immature nucleus compared to the diabetic group (p < 0.001). There was no significant effect in the sperm maturation process in the GL- treated animals compared to diabetic group (p = 0.387). GL administration had no significant effect on sperm maturation process in comparison with diabetic group (p = 0.387).

### MDA measurement in serum

According to the results in table I, the plasma level of MDA was remarkably increased in diabetic rats compared to the control group (p < 0.001). Administration of GL and TR was considerably reduced the concentration of MDA in plasma (p < 0.001) compared to the diabetic group. Also, a significant difference was seen between GL and TR groups in terms of plasma level of MDA. Furthermore, there was a significant difference between (GL+TR) with GL and TR groups (p < 0.001).

### TAC measurement in serum

Serum level of TAC decreased significantly in the diabetic rats compared to the control group (p < 0.001) (Table I). Statistical analysis revealed that, treatment with GL and TR was considerably increased the concentration of TAC in serum compared to the diabetic group (p < 0.001). Also, the mean concentration of TAC showed a significant rise in TR group as compared with the GL group (p < 0.001). In addition, a significant difference was observed between (GL+TR) with GL (p < 0.001) and TR (p = 0.004) groups in terms of MDA concentration.

### Body weight and testis weight

Table II presented the effects of various agents on the rat's body and testis weight and index of testis weight. According to the results there was no significant difference between the experimental groups in terms of initial body weight (p = 0.622. At the end of the study, a considerable lower body weight was observed in the diabetic group compared to the control group (p < 0.001). A partial improvement in body weight was seen in animals treated with GL, TR, and their combination.

A significant difference was observed in terms of final body weight between diabetic and treated animals (p < 0.001), between GL and TR groups (p < 0.001), and between (GL+TR) with GL and TR groups (p < 0.001).

A considerable lower testis weight was seen in the diabetic group compared to the control group (p < 0.001). Findings of the study indicated that treatment with GL and TR resulted in a significant increase in testis weight (p < 0.001) compared to diabetic group. Furthermore, a significant difference was seen between GL and TR groups in terms of testis weight (p = 0.038). Also, a significant difference was observed between (GL+TR) with GL (p < 0.001) and TR (p ≤ 0.001) groups.

### Effect of GL and TR on seminiferous tubules diameter and germinal epithelium height 

**Figure 1:**
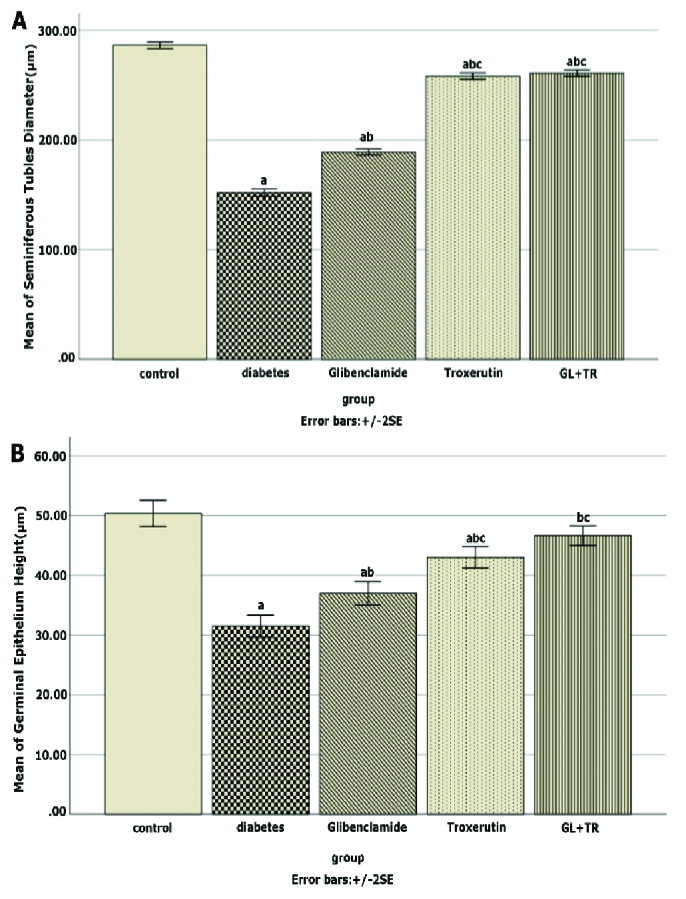
Effect of glibenclamide (GL) and troxerutin (TR) on A) Seminiferous tubules diameter and B) Germinal epithelium height in different groups. The data were expressed as Mean ± SE. a: Significant difference from the control group, b: Significant difference from the diabetic group, c: Significant difference with GL group.

Figure 1 presents seminiferous tubules' diameter and germinal epithelium's height in the cross sections of the testis for all experimental groups. Compared to the control group, DM reduced seminiferous tubules diameter significantly (p < 0.001), while in the animals treated with GL and TR, this parameter's mean was significantly higher than that of the diabetic rats (p < 0.001). Moreover, there was a significant difference between GL and TR groups in terms of seminiferous tubules diameter (p < 0.001). The height of the germinal epithelium was significantly decreased in the diabetic group compared to the control group (p < 0.001). Treatment with GL (p = 0.002) and TR (p < 0.001) significantly increased the thickness of the germinal epithelium in comparison with the diabetic group. Further, a significant difference was seen in terms of germinal epithelium height between (GL and TR groups (p ≤ 0.001)).

### Effect of GL and TR on spermatogenic cells' count

**Figure 2:**
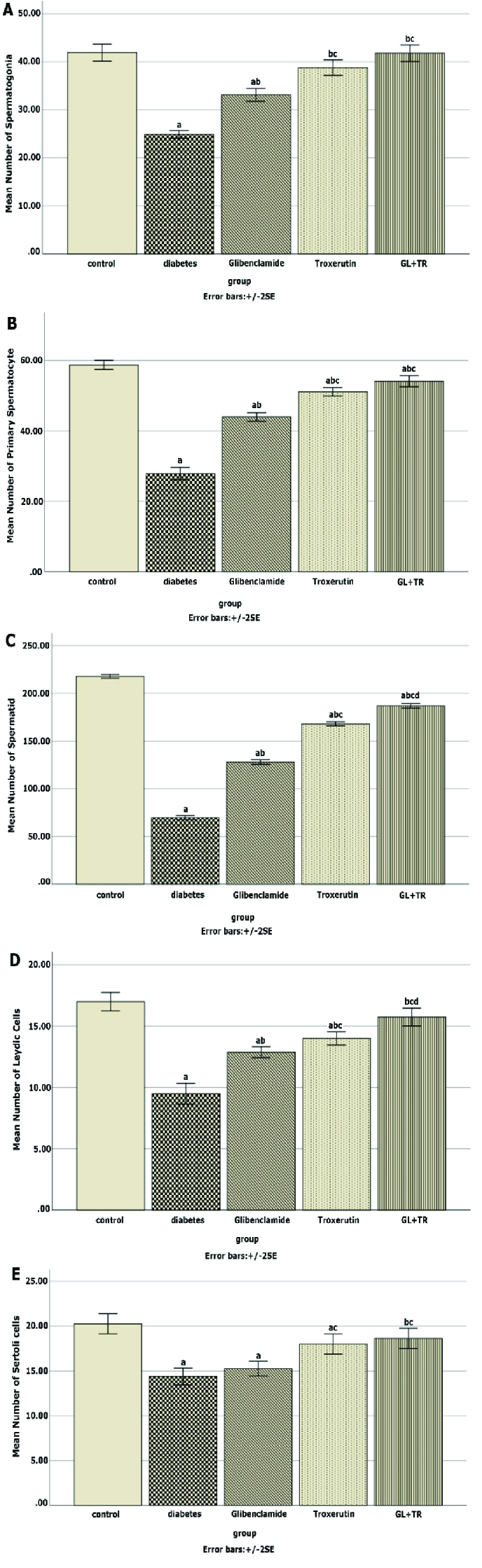
Effect of glibenclamide (GL) and troxerutin (TR) on the number of A) Spermatogonia, B) Primary spermatocyte, C) Spermatids, D) Leydig cells, and E) Sertoli cells, in different groups. Data were expressed as Mean ± SE. a: Significant difference with the control group, b: Significant difference with the diabetic group, c: Significant difference with GL group, and d: Significant difference with TR group.

Figure 2 depicts the average number of spermatogenic cells. According to figure 2, the diabetic group had a significantly lower number of spermatogonia, primary spermatocytes, spermatid, Leydig cells, and Sertoli cells than the control group (p < 0.001). Treatment with GL and TR significantly increased the above-mentioned cell count (p < 0.001).

### Histopathological findings

**Figure 3:**
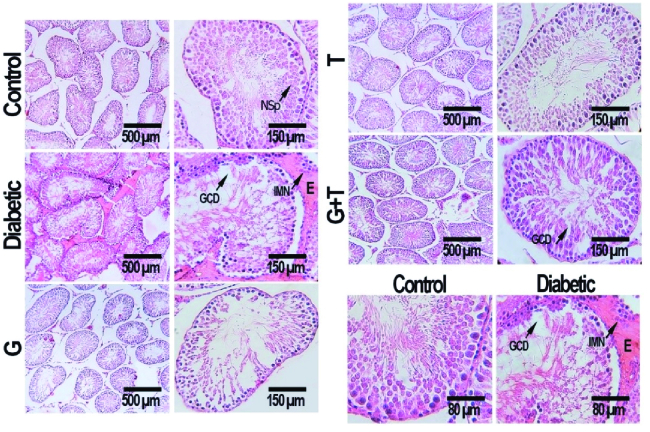
Cross sections of testicular tissue from different groups, see normal cell distribution and normal cell spermatogenesis (NSp) in the control group. The section from the diabetes group shows germinal epithelium height reduction, incomplete spermatogenic series, germinal cells dissociation (GCD), and irregular germ epithelium arrangement, edema (E), and immune cells infiltration (IMN), which are ameliorated in the GL, TR, and GL+TR groups (H&E, 200x, 600x, and 800x).

Histopathological analysis of the testis tissue revealed that the morphology of seminiferous tubules was normal in the control group, and the germinal epithelium had a normal height and contained all spermatogenic cells, such as spermatogonia, primary spermatocytes, and spermatids. A large number of spermatozoids were seen in the lumen of seminiferous tubules. The thickness of the basement membrane was normal, and the interstitial spaces contained several Leydig cells. Disorganization and deformation of seminiferous tubules, degeneration of germinal epithelium and atrophy of Leydig cells were observed in the diabetic group. GL and TR treatment decreased diabetic histopathologic problems considerably (Figure 3).

### Apoptosis assay

**Figure 4:**
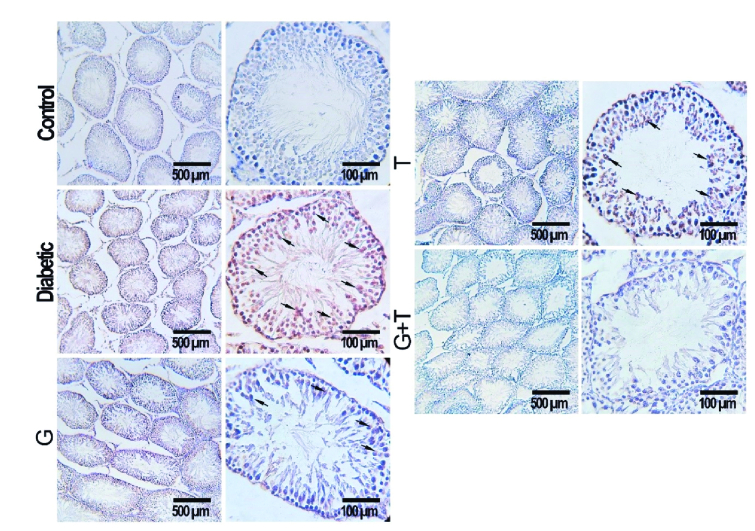
Effect of glibenclamide and troxerutin on spermatogenic cells' apoptosis in different groups. Apoptotic cells are turning dark brown (black arrow). GL: Glibenclamide, TR: Troxerutin (TUNEL staining, 200x, 600x).

**Figure 5:**
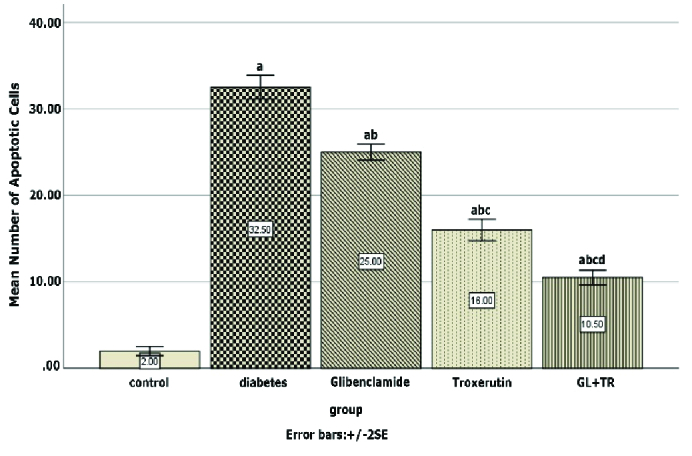
Effect of glibenclamide and troxerutin on apoptosis of spermatogenic cells in experimental groups. a: Significant difference with the control group, b: Significant difference with the diabetic group, c: Significant difference with GL group, and d: Significant difference with TR group.

Figure 4 represents the presence of the apoptotic cells in seminiferous tubules of rats in experimental groups. The number of apoptotic cells in the control group was negligible, while they were observed in the seminiferous tubules of other experimental groups (Figure 4). According to the statistical analysis, animals in the diabetic group showed a significant increase in apoptotic cells compared to the control group (p < 0.001). Rats treated with GL and TR and their combination showed a remarkable decrease in the number of TUNEL-positive cells compared to the diabetic group (p < 0.001). Moreover, a significant difference was observed in terms of TUNEL-positive cells between GL and TR groups (p < 0.001) (Figure 5). Also, there was a significant difference between (GL+TR) with GL and TR groups (p < 0.001). The DFI was calculated for each experimental group and was presented in table I.

**Table 1:** Effects of GL and TR on sperm parameters


**Variable**	**Control**	**Diabetes**	**GL**	**TR**	**GL+TR**	**P-value**
**Sperm count (ml × 10^6^)**	119.75 ± 2.92 (1.03)	37 ± 3.16 ${}^{\text{a}}$ (1.12)	51 ± 2.33 ${}^{\text{ab}}$ (0.82)	78.25 ± 2.96 ${}^{\text{abc}}$ (1.05)	91.25 ± 3.01 ${}^{\text{abcd}}$ (1.06)	< 0.001
**Motile sperms (%)**	64 ± 3.25 (1.15)	23.13 ± 2.80 ${}^{\text{a}}$ (0.99)	47.50 ± 2.67 ${}^{\text{ab}}$ (0.94)	57.25 ± 2.82 ${}^{\text{abc}}$ (1)	60.25 ± 2.49 ${}^{\text{bc}}$ (0.88)	< 0.001
**Sperm with normal** **morphology (%)**	87 ± 2.78 (0.98)	46.88 ± 2.42 ${}^{\text{a}}$ (0.85)	68 ± 3.16 ${}^{\text{ab}}$ (1.12)	78.38 ± 2.13 ${}^{\text{abc}}$ (0.75)	83.13 ± 2.42 ${}^{\text{bcd}}$ (0.85)	< 0.001
**Viability of sperm (%)**	90.25 ± 2.87 (1.01)	43.13 ± 2.59 ${}^{\text{a}}$ (0.91)	57.63 ± 2.83 ${}^{\text{ab}}$ (1)	70.13 ± 3.04 ${}^{\text{abc}}$ (1.08)	88.25 ± 2.66 ${}^{\text{bcd}}$ (0.94)	< 0.001
**Sperm with immature** **nucleus (%)**	7.75 ± 1.39 (0.49)	41.25 ± 4.30 ${}^{\text{a}}$ (1.52)	38.38 ± 2.13 ${}^{\text{a}}$ (0.75)	31 ± 2.33 ${}^{\text{abc}}$ (0.82)	28.63 ± 2.33 ${}^{\text{abc}}$ (0.82)	< 0.001
**MDA (nmol/ml)**	1.4 ± 0.14 (0.05)	4.84 ± 0.18 ${}^{\text{a}}$ (0.06)	3.80 ± 0.16 ${}^{\text{ab}}$ (0.06)	2.30 ± 0.16 ${}^{\text{abc}}$ (0.06)	1.60 ± 0.23 ${}^{\text{bcd}}$ (0.08)	< 0.001
**TAC (nmol/ml)**	0.84 ± 0.11 (0.04)	0.27 ± 0.02 ${}^{\text{a}}$ (0.01)	0.42 ± 0.02 ${}^{\text{ab}}$ (0.01)	0.60 ± 0.03 ${}^{\text{abc}}$ (0.01)	0.71 ± 0.04 ${}^{\text{abcd}}$ (0.01)	< 0.001
**DFI (%)**	1.66 ± 0.63 (0.22)	27.8 ± 1.60 ${}^{\text{a}}$ (0.57)	20.83 ± 1.09 ${}^{\text{ab}}$ (0.39)	13.33 ± 1.48 ${}^{\text{abc}}$ (0.52)	8.75 ± 1.00 ${}^{\text{abc}}$ (0.35)	< 0.001
Data were expressed as Mean ± SD (SE). One way-ANOVA test. MDA: Malondialdehyde, TAC: Total antioxidant capacity, DFI: DNA fragmentation index, GL: Glibenclamide, TR: Troxerutin. a: Significant difference with the control group, b: Significant difference with the diabetic group, c: Significant difference with GL group, and d: Significant difference with TR group						

**Table 2:** Effects of GL and TR on body and testis weights and index of testis weight in different groups


**Variable**	**Control**	**Diabetes**	**GL**	**TR**	**GL+TR**	**P-value**
Initial body weight (g)*	254.63 ± 7.11 (2.51)	256.13 ± 7.97 (2.82)	255.25 ± 7.23 (2.55)	253.13 ± 6.62 (2.34)	258.63 ± 6.35 (2.24)	0.622
Final body weight (g)*	266.38 ± 8.11 (2.87)	157.25 ± 7.85 ${}^{\text{a}}$ (2.78)	182.13 ± 5.99 ${}^{\text{ab}}$ (2.12)	214.75 ± 4.37 ${}^{\text{abc}}$ (1.54)	243.38 ± 6.41 ${}^{\text{abcd}}$ (2.27)	< 0.001*
Testis weight (g)*	1.70 ± 0.05 (0.02)	0.92 ± 0.04 ${}^{\text{a}}$ (0.01)	1.24 ± 0.17 ${}^{\text{ab}}$ (0.06)	1.37 ± 0.02 ${}^{\text{abc}}$ (0.01)	1.55 ± 0.04 ${}^{\text{abcd}}$ (0.02)	< 0.001*
Index of testis weight (%)**	0.0064 (0.64 %)	0.0059 (0.59 %)	0.0068 (0.68 %)	0.0064 (0.64 %)	0.0064 (0.64 %)	-
*Data were expressed as Mean ± SD (SE). **Data presented as weight (%). One way-ANOVA test. GL: Glibenclamide, TR: Troxerutin, a: Significant difference with the control group, b: Significant difference with the diabetic group, c: Significant difference with GL group, d: Significant difference with TR group						

## 4. Discussion

The present study aimed to compare the effects of TR with GL on the testis and sperm parameters in diabetic rats. Body and testis weight, sperm parameters, sperm DNA integrity, seminiferous tubule diameter, the number of spermatogenic cells, and the TAC level were dramatically decreased, while MDA levels and the number of apoptotic cells in the testicular tissue were increased in diabetic animals in contrast to the control group. The use of TR and GL markedly improved the above-mentioned parameters (p < 0.001). The findings of this study are consistent with previous researches.

A previous study investigated the effect of TR on male fertility in diabetic male rats. Its findings indicated that DM caused a significant reduction in glutathione peroxidase (GPx), testosterone, the total number, viability, and motility of sperm, and treatment with TR significantly improved the above-mentioned parameters (24).

This study indicated that DM decreased the levels of sperm parameters, including the sperm count, motility, viability, and normal morphology. High blood sugar levels or hyperglycemia is one of the major factors that affect fertility (25). Previous study has suggested that hyperglycemia induces an excessive production of ROS and reactive nitrogen species (26).

Hyperglycemia-induced OS has negative consequences on the reproductive system, including body weight loss and sexual organ damage. It also causes testicular atrophy and a reduction in total sperm count (27). The cell membrane of sperm contains a high concentration of polyunsaturated fatty acids, making it very sensitive to OS; thus, lipid peroxidation defects the integrity of the sperm cell membrane, causing a decrease in sperm motility and viability, eventually leading to apoptosis and a decrease in sperm count. Antioxidant supplementation can protect the sperm against ROS-induced DNA damage and improve sperm quality, therefore, antioxidant administration could be one of the beneficial therapeutic methods for preventing diabetic-induced infertility.

TR, which is commonly known as vitamin P4, is a derivative of bioflavonoids that possess several biological and pharmacological properties such as antioxidant (7), antihyperglycemic (8), and anti-inflammatory effects (9). The antioxidant properties of TR may be due to its impact on reducing ROS production and activating antioxidant enzymes (11, 14). Moreover, TR increases cell sensitivity to insulin (7, 12).

GL is a long-used agent which is administered in patients with non-insulin-dependent DM. Previous studies have suggested that GL can counteract ROS production and prevent OS (15).

Another study examined the effect of TR on nickel-induced testicular toxicity in Wistar rats and revealed that TR administration could reverse this toxicity (16). TR boosted testis weight while lowering OS levels. Previous research has shown that DM-induced OS reduces the amounts of antioxidant enzymes in Leydig cells, lowering testosterone production. Low testosterone levels results in spermatogenesis dysfunction and degeneration of seminiferous tubules and eventually reduction of the testis weight. In this study, the administration of TR and GL, increased serum testosterone level which led to an improvement in count and function of germinal cells. Finally, it led to an increase in testicular weight.

The effects of GL and losartan on diabetic rats was investigated by researchers (28). They reported that DM considerably increased systemic hepatic and renal inflammation and decreased antioxidant defense system. Administration of GL or losartan significantly improved hepatic and renal functions and their histological structure. Furthermore, they confirmed that both agents attenuated the signs of OS and inflammation in the blood, liver, and kidney samples.

In recent research on diabetic adult rats, the effects of TR and insulin on testicular function and structure were investigated (14). Its findings demonstrated that DM elevated blood glucose levels, testicular MDA, and apoptosis substantially. It also lowered sperm parameters, insulin levels, and testicular GPx activity, as well as causing stereological changes. They reported that the administration of TR significantly improved the above-mentioned parameters. They concluded that TR has anti-apoptotic effects and efficiently improved diabetic-induced testicular dysfunction and sperm parameters compared to insulin.

These findings were in accordance with those of the present study. The data of the present study revealed that DM significantly increased the serum level of MDA and apoptosis in spermatogenic cells and caused morphometric and stereological changes, such as a reduction in the number of spermatogonia, spermatocytes, spermatids, Leydig, and Sertoli cells. It also decreased the serum level of TAC. Treatment with TR and GL markedly improved the above-mentioned parameters. The findings also showed that the TR effects were comparable to those of GL.

Evidence has strongly confirmed that DM increases OS and apoptosis whereas it decreases sperm count, motility and viability and the number of Leydig and Sertoli cells and eventually lead to infertility (25). Similarly, the present investigation showed that the levels of OS and lipid peroxidation were higher in diabetic rats than the control group. Other studies have indicated that diabetes decreases the activity of antioxidant enzymes such as GPx (29). A recent study indicated that DM lowers serum TAC levels. The fact that DM lowers antioxidant defense responses were validated by this research. As a result of these processes, diabetes changes sperm parameters and reduces the amount of Leydig and Sertoli cells. Researchers (30) studied the effect of TR on OS in experimental model of sciatic nerve ischemia-reperfusion injury. They reported that administration of TR leads to an increase in GPx and paraoxonase 1 levels and concluded that TR increased the levels of antioxidant enzymes in sciatic nerve ischemia-reperfusion injury and attenuated the OS. The different properties of TR, including antihyperglycemic, anti-inflammatory, antioxidant, and antiapoptotic effects, have been reported by several investigations (6, 8, 10).

The present study showed that TR significantly improved the number of spermatogenic cells such as spermatogonia, spermatocyte, spermatids, Leydig, and Sertoli cells in the diabetic group. This had better effect than GL. This agent's antiapoptotic properties might explain TR's above-mentioned positive effects. This study's findings showed that TR markedly reduced the number of TUNEL-positive cells in seminiferous tubules of diabetic rat testes. These findings were in accordance with those of previous studies (14).

The effect of TR on Alzheimer's disease was investigated by other researchers (31). Their results showed that ICV (intracerebroventricular) microinjection of amyloid-beta increased the MDA level and reduced superoxide dismutase and GPx activities in the hippocampus. In the hippocampus, TR treatment dramatically reduced MDA levels while increasing superoxide dismutase and GPx activity. Furthermore, TR treatment reduced the number of TUNEL-positive apoptotic cells in the dentate gyrus. They concluded that TR had an antiapoptotic effect. In a previous study researchers induced the experimental model of myocardial ischemia-reperfusion injury in diabetic rats, and investigated the effect of TR on the phosphorylation of GSK-3β protein and apoptosis (32). Its findings showed that TR increased the level of the phosphorylated form of GSK-3β in diabetic groups. Moreover, TR significantly decreased the apoptotic tissue level and apoptotic index. The authors explained that the phosphorylation of GSK-3β could be a mechanism by which TR attenuated the apoptotic level.

The data of present study showed that administration of TR led to significant improvements in sperm parameters, the number of spermatogenic cells, and biochemical parameters in diabetic rats compared with the GL-treated group. Another study (33) examined the antioxidant effect of ginger and GL in STZ-induced diabetic rats. The results revealed that both ginger and GL have antioxidant effects and free radical scavenging properties, but ginger is more capable of eliminating them. The current study showed that both TR and GL are scavengers of free radicals, but TR is more capable than GL in eliminating free radicals.

## 5. Conclusion

This study's findings revealed that TR markedly improves sperm parameters and diabetic-induced testicular dysfunction and this effect was superior to those of GL. Its antiapoptotic properties could explain the beneficial effect of TR. In summary, this study confirmed that TR as an herbal agent possesses the potential to be used to treat diabetic-induced testicular dysfunction and infertility.

##  Conflict of Interest

The authors declare no conflict of interest in the present study. 
